# Treatment for Stanford type B aortic dissection with insufficient anchoring region using castor integrated branched aortic stent graft

**DOI:** 10.3389/fcvm.2024.1351342

**Published:** 2024-03-25

**Authors:** Weiqing Chen, Dabing Liu, Tao Chen, Jian Liu, Yi Guo, Bo Ye

**Affiliations:** ^1^ Department of Vascular Surgery, Ganzhou People’s Hospital, Ganzhou, Jiangxi, China; ^2^ Department of General Surgery, The People’s Hospital of Ganxian District, Ganzhou, Jiangxi, China

**Keywords:** integrated branched stent, aortic dissection, thoracic endovascular aortic repair (TEVAR), anchoring region, castor

## Abstract

**Background:**

To investigate the clinical efficacy of Castor integrated branched aortic stent graft for the treatment of Stanford type B aortic dissection with insufficient anchoring area.

**Methods:**

Retrospective analysis of clinical data of 26 patients with Stanford type B aortic dissection with insufficient anchoring region (<15 mm) treated by Castor branched aortic stent graft from September 2018 to June 2022 at Ganzhou People's Hospital, including 23 acute cases and 3 chronic cases.

**Results:**

Surgical procedures were successfully performed in all 26 patients, and during the perioperative period no complications occurred, such as cerebrovascular accident, stenosis or occlusion of left subclavian artery, progression of reverse avulsion of aortic dissection, and paraplegia. During the operation 2 patients had a small amount of type I endoleak, which disappeared during the postoperative follow-up. The other patients had good postoperative follow-up results. Review of the aortic CTA indicated good stent morphology with patency of the left subclavian artery.

**Conclusions:**

The Castor integrated branched aortic stent graft expanded the indications for endoluminal treatment for Stanford type B aortic dissection, which can avoid open surgery and has good clinical outcomes.

## 1 Introduction

Aortic dissection, a highly dangerous medical condition, is a common emergency in vascular surgery. With rapid development of endovascular technology and related equipment, thoracic endovascular aortic repair (TEVAR) has become the first choice for the treatment of Stanford B aortic dissection (Type B aortic dissection, TBAD) because of its minimal trauma, quick recovery, good therapeutic effect and fewer complications (1). In normal circumstances, the proximal healthy anchoring zone is required to be longer than 15 mm, so that TEVAR can be performed with conventional aortic covered stent (2, 3). Insufficient proximal healthy anchoring area (<15 mm) may lead to the reverse avulsion of aortic dissection, endoleak and occlusion of aortic branches. Therefore, when the healthy anchoring area is shorter than 15 mm, the proximal anchoring area should be expanded in endovascular surgery, mostly around the left subclavian artery (LSA). Techniques such as carotid-subclavian or axillary-axillary artery artificial vascular bypass, parallel stent technique, fenestration technique, LSA endovascular reconstruction can be adopted, with the main purpose of providing sufficient healthy anchoring area for the covered stent and keeping the LSA unobstructed. In recent years, our department has adopted the Castor integrated branched aortic covered stent to treat Stanford B aortic dissection with insufficient anchoring area (4, 5), which is reported as follows.

## 2 Materials and methods

### 2.1 The research object

We retrospectively analyzed the clinical data of Stanford type B aortic dissection patients with insufficient anchoring zone treated by Castor integrated branched aortic covered stent in Ganzhou People's Hospital from September 2018 to September 2022. All patients in this group were examined by CTA before operation, and the diagnosis of Stanford B aortic dissection was confirmed, with healthy anchoring area shorter than 15 mm (the distance from the rupture of aortic intima or the avulsion dissection to the opening of LSA).

### 2.2 Pre-operative preparation

**Figure 1:**
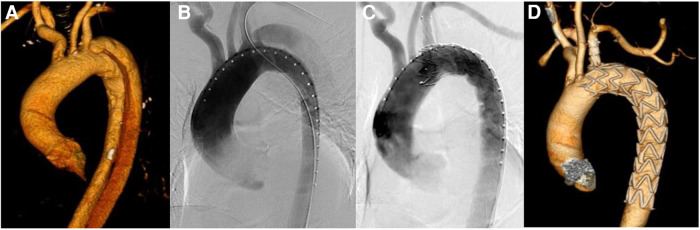
Results of aortic imaging in patients at different periods. (**A**) Is the preoperative CTA image of aorta. (**B**) Is the result of DSA angiography of aorta during operation, and the intimal rupture is visible near the root of LSA. (**C**) Is the result of DSA after the release of Castor integrated branched aortic covered stent. The intimal rupture is closed, with no visible endoleak, and LSA is unobstructed. (**D**) Is CTA imaging review of aorta more than one month after operation, which shows that the stent is well extended, the original false lumen is thrombed and LSA is unobstructed.

Acute cases in this group were demanded strict confinement in bed and given active pain relieving after admission; Strict control of blood pressure and heart rate: blood pressure was quickly brought down below 120/80 mmHg (1 mmHg = 0.133 kPa) and heart rate maintained at 60∼70 bpm. Close observation of patient's state of mind, vital signs, skin temperature of limbs, pain changes, urine volume, existence of dyspnea or displegia, smooth defecation, etc. CTA for the aorta, ECG, color Doppler echocardiography and biochemical tests were performed for all patients in this group before operation. The aortic diameter at the opening of LSA, LSA diameter, descending aorta diameter, the length of healthy anchoring zone and the length from the left common carotid artery to LSA were measured and preoperative evaluation was actively completed (Figure 1A). Covered stents with 10%–20% oversize in diameter, and branch stents with 1–2 mm enlargement were often chosen for the procedure.

### 2.3 Operation methods

Patients in this group were given general anesthesia and endotracheal intubation. Inguinal region and left elbow incision were taken to expose the femoral artery and the left brachial artery, and heparin sodium was injected intravenously for systemic heparinization (0.5 mg/kg). Vascular sheath was inserted via femoral artery access, and gold-labeled pig tail catheter were deployed into ascending aorta through the descending aorta true lumen, and the location of LSA opening were confirmed by angiography (Figure 1B). Seldinger technique was used to puncture the left brachial artery under direct vision, and a 7F vascular sheath was inserted. Through this vascular sheath, a catheter guidewire was inserted and led out of the body from the femoral artery vascular sheath through the descending aorta, and the guidewire was removed while the catheter remained in the blood vessel. Lunderquist guide wire was placed in ascending aorta through femoral artery gold-labeled pig tail catheter, and the gold-labeled pig tail catheter was withdrawn. After air expulsion, Castor integrated branched aortic covered stent was deployed through Lunderquist guide wire of femoral artery. At the mean time, the branched guide wire was placed in femoral artery catheter and led out of the body from the left brachial artery vascular sheath, and the stent body was placed along Lunderquist guide wire. Meanwhile, the branched guide wire was pulled, and the conical head of the delivery system was led into the upper segment of descending aorta. Adjust the 8-shaped marker at the head of the soft sheath to the lesser curvature side of the aortic arch under fluoroscopy, and transform the marker into an I-shape. Continue to push the distal end of the conical head of the delivery system to the same height of LSA opening, and pay attention to avoid entanglement between the branch guide wire and the delivery system. If entanglement occurs, the delivery system needs to be withdrawn to the straight section of the descending aorta and then the handle of the outer tube needs to be rotated to unwind the entanglement. Retract the soft sheath which wrapped around the stent to the limit position, to expose the main stent and the branch stent, then continue to push the stent body and pull the branch guide wire at the same time, so that the branch stent can be placed in LSA. Confirm that the branch stent is located on the greater aortic curvature side. Fine-tune the position of the main body up and down to make the LSA branch stent completely attach to the LSA blood vessel wall. Pull the branch guide wire and stabilize the delivery system, and release the stent body. After the main stent is released, pull the branch guide wire and catheter to release the branch stent. Perform another angiography to clarify the stent position, whether there is endoleak and the patency of LSA branch stent (Figure 1C). Withdraw the arterial sheath and catheter, suture the puncture of femoral artery and left brachial artery, and suture each surgical incision layer by layer.

### 2.4 Follow-up and observation indicators

Patients were routinely followed up 1 month, 3 months, 6 months and every year after discharge. At each follow-up, CTA imaging of aorta were performed, blood pressure and heart rate were monitored and discomforts with left upper limb, head, chest or back were recorded. CTA examination of aorta confirmed the stent's position and shape, existence of endoleak, patency of branch stents, false lumen thrombosis, etc.

### 2.5 Statistical analysis

Spss 25.0 statistical software was used, and the measurement data were represented as *X* ± *S*. Paired T test was used to compare the changes of true and false aortic lumen diameters at different levels before and one year after TEVAR, and *P* < 0.05 indicated that the difference was statistically significant.

## 3 Results

26 patients were included in this group, including 20 males and 6 females. There were 13 cases of coronary heart disease, 5 cases of diabetes, 3 cases of chronic renal insufficiency and 8 cases of chronic obstructive pulmonary disease. 17 cases had a history of smoking. All the 26 patients were successfully operated. During the operation, 2 patients had a small amount of type I endoleak, and there were no perioperative complications such as cerebrovascular accident, left subclavian artery stenosis or occlusion, aortic dissection and paraplegia. In this group, CTA of aorta was reviewed 1 month, 3 months, 6 months and every year after operation. The total follow-up duration was 12–48 months, with an average of 24 ± 6.4 months. Two patients had a small amount of type I endoleak, which disappeared in subsequent follow-ups. The follow-ups of the other 24 patients all showed satisfactory results. The CTA of aorta showed that the stent was in good shape, the left subclavian artery was unobstructed, and the false lumen covered by the original thoracic aorta stent was absorbed after thrombosis. The changes of true and false lumen diameters of each segment of aorta before and 1 year after operation are shown in Table 1.

**Table 1:** Changes of true and false lumen diameters in 26 patients before and 1 year after operation.

Level		Before operation (*x* ± *s*, mm)	1 year after operation (*x* ± *s*, mm)	*P* value
Pulmonary artery bifurcation level	Ture lumen	7.9 ± 3.9	30.3 ± 4.7	<0.05
	False lumen	25.9 ± 6.1	2.1 ± 0.5	<0.05
The distal end of the stent level	Ture lumen	7.2 ± 3.5	23.9 ± 3.2	<0.05
	False lumen	18.9 ± 3.9	3.9 ± 0.7	<0.05

## 4 Discussion

At present, TEVAR is the first choice for the treatment of TBAD. When the proximal healthy anchoring zone is shorter than 15 mm, the use of conventional aortic covered stent will cause partial or complete occlusion of LSA, which will lead to LSA steal syndrome, higher risk of vascular injury, stent displacement and endoleak (3, 6–8). LSA provides extensive blood supply to the brain, left upper limb and spinal cord. If LSA is completely blocked during TEVAR operation, it will increase the risk of postoperative complications such as insufficient blood supply to left upper limb artery and vertebrobasilar artery. In severe cases, stroke, paraplegia, upper limb ischemia and necrosis are also likely to occur (9–11). Therefore, in 2009, the American Society of Vascular Surgery issued guidelines recommending LSA revascularization be performed whenever possible (7). It is now considered that keeping LSA unobstructed can reduce postoperative complications and improve long-term prognosis of TEVAR (12).

Currently, there are several approaches to reconstruct LSA during TEVAR, such as hybrid procedure, parallel stent technique, fenestration technique, single-branch stent technique, etc. Hybrid techniques, such as carotid-subclavian or axillary-axillary artery artificial vascular bypass, can rebuild LSA blood supply by external anatomical means, which requires fine surgical skills of the operator to avoid surgery-related complications, such as bleeding, neurolymphatic injury, infection, etc. (13). Parallel stent technique: it includes the anterograde chimney technique and the retrograde periscope technique, and the chimney technique is more commonly used at present. The operation of this technique is relatively simple, but branched vascular stents and aortic covered stents are placed in parallel in the aorta, and there is a greater possibility of endoleak due to the gap between stents (14). In addition, the blood flow pattern at the parallel stent is abnormal, which tends to form blood vortex and cause thrombosis of the branch stent (15). Fenestration techniques: mainly includes in-vitro pre-fenestration and in-situ fenestration. In in-vitro pre-fenestration procedures, the operater is required to make accurate measurement according to CTA results before operation, open the covered stent *in vitro*, and make a hole with the same size as the LSA in the corresponding position of the covered stent in advance. When the stent is released, align the hole with the LSA opening to rebuild the LSA. In-situ fenestration is to cover the LSA opening when the aortic covered stent is placed, puncture the left brachial artery, and with the assistance of vascular sheath, use puncture needle or laser to fenestrate on the aortic covered stent, place the stent, and rebuild LSA. Because LSA is reconstructed through anatomical approach, the original hemodynamic characteristics can be preserved. This technique is widely used in clinical practice due to its minimal trauma and low cost, but it also has disadvantages because of its complicated operation, high requirments for skills, and increased risk of endoleak due to the damage of integrity and stability of the stent (16). Single-branch stent technique: Castor integrated branched aortic covered stent is the first branched covered stent approved for clinical use in China. The integrated design makes the main body and branches firmly anchored in the vascular lumen, which is more in line with the anatomical and physiological characteristics of human blood vessels, and can reduce the risk of stent displacement and endoleak and improve the patency rate of stent blood vessels (17). Castor integrated branched aortic covered stent is reasonable in design and simple in operation. There are four specifications for the covered length from the front end of the main stent to the beginning of the branch: 5 mm, 10 mm, 15 mm and 30 mm, which is suitable for the interval between most LSAs and the left common carotid artery. In addition, the Castor integrated branched aortic covered stent has a vertebral body design, which can better adapt to various aortic shapes and reduce the risk of endoleak. In this study, there were 2 cases with a small amount of type I endoleak, which disappeared in subsequent follow-ups. That might be due to further adaptation of the stent to the blood vessel wall in postoperative remodeling. In this group of patients, the first intima of aorta was well isolated during the operation. During the follow-ups, results show that the true lumen of aorta increased, the false lumen decreased and thrombed after TEVAR, and the changes in true and false lumen diameter were statistically significant. During the release process of Castor integrated branched aortic covered stent, attention should be paid to the following issues: (1) Both the main body of covered stent and the guide wire of branched stent should be in the aortic lumen. (2) If the branched guide wire is entangled with the delivery system during the operation, the delivery system should be withdrawn to the straight segment of descending aorta for unwinding. (3) If stenosis exists after the branch stent is released, balloon expansion can be applied, and stents can be placed, if necessary, to keep the LSA unobstructed.

Castor integrated branched aortic covered stent entered the market in 2017, and has not been in use for quite long. However, based on our experience in treating this group of cases, we found that this stent is easy to operate, stable after release, with less risk of endoleak and higher rate of LSA patency. The short-term follow-ups showed good results, but long-term prognosis needs further follow-ups (5, 18).

To sum up, in the treatment of TBAD with insufficient proximal healthy anchoring zone, Castor integrated branched aortic covered stent effectively prolongs the healthy anchoring zone and expands the surgical indications of TEVAR. This technique reconstructs LSA and maintains the stability of the stent when treating aortic dissection, thus open surgery is avoided. The outcome is satisfactory.

## 5 Conclusion

In the treatment of TBAD with insufficient proximal healthy anchoring zone, Castor integrated branched aortic covered stent effectively prolongs the healthy anchoring zone and expands the surgical indications of TEVAR.

## Data Availability

The raw data supporting the conclusions of this article will be made available by the authors, without undue reservation.

## References

[1] Parsa CJ Williams JB Bhattacharya SD Wolfe WG Daneshmand MA McCann RL Midterm results with thoracic endovascular aortic repair for chronic type b aortic dissection with associated aneurysm. J Thorac Cardiovasc Surg. (2011) 141(2):322–7. 10.1016/j.jtcvs.2010.10.043 21241855 PMC3688644

[2] Chen S Larion S Ahanchi SS Ammar CP Brandt CT Panneton JM. Erratum to: A novel anatomic severity grading score for acute type B aortic dissections and correlation to aortic reinterventions after thoracic endovascular aortic repair. Cardiothorac Surg. (2017) 12(1):53–6. 10.1186/s13019-017-0616-2 PMC5482932 28645316

[3] Bradshaw RJ Ahanchi SS Powell O Larion S Brandt C Soult MC Left subclavian artery revascularization in zone 2 thoracic endovascular aortic repair is associated with lower stroke risk across all aortic diseases. J Vasc Surg. (2017) 65(5):1270–9. 10.1016/j.jvs.2016.10.111 28216353

[4] Lu Q Feng J Zhou J Zhao Z Li H Teng Z Endovascular repair by customized branched stent-graft: a promising treatment for chronic aortic dissection involving the arch branches. J Thorac Cardiovasc Surg. (2015) 150:1631–8. 10.1016/j.jtcvs.2015.08.032 26384748

[5] Jing Z Lu Q Feng J Zhou J Feng R Zhao Z Endovascular repair of aortic dissection involving the left subclavian artery by castor stent graft: a multicentre prospective trial. Eur J Vasc Endovasc Surg. (2020) 60(6):854–61. 10.1016/j.ejvs.2020.08.022 33183920

[6] Waterford SD Chou D Bombien R Uzun I Shah A Khoynezhad A. Left subclavian arterial coverage and atroke during thoracic aortic endografting: a systematic review. Ann Thorac Surg. (2016) 101(1):381–9. 10.1016/j.athoracsur.2015.05.138 26588864

[7] Matsumura JS Lee WA Mitchell RS Farber MA Murad MH Lumsden AB The society for vascular surgery practice guidelines: management of the left subclavian artery with thoracic endovascular aortic repair. J Vasc Surg. (2009) 50(5):1155–8. 10.1016/j.jvs.2009.08.090 19878791

[8] Mesar T Alie-Cusson FS Rathore A Dexter DJ Stokes GK Panneton JM. A more proximal landing zone is preferred for thoracic endovascular repair of acute type B aortic dissections. J Vasc Surg. (2022) 75(1):38–46. 10.1016/j.jvs.2021.06.036 34197944

[9] Fang C Wang C Liu K Pang X. Early outcomes of left subclavian artery revascularization using castor single-branched stent-graft in the treatment of type B aortic dissection or intramural hematoma. Ann Thorac Cardiovasc Surg. (2021) 27(4):251–9. 10.5761/atcs.oa.20-00166 PMC8374093 33342930

[10] Zamor KC Eskandari MK Rodriguez HE Ho KJ Morasch MD Hoel AW. Outcomes of thoracic endovascular aortic repair and subclavian revascularization techniques. J Am Coll Surg. (2015) 221(1):93–100. 10.1016/j.jamcollsurg.2015.02.028 PMC4478203 25872688

[11] Riambau V Böckler D Brunkwall J Cao P Chiesa R Coppi G Editor’s choice-management of descending thoracic aorta diseases: clinical practice guidelines of the European society for vascular surgery (ESVS). Eur J Vasc Endovasc Surg. (2017) 53(1):4–52. 10.1016/j.ejvs.2016.06.005 28081802

[12] Awad H Ramadan ME El Sayed HF Tolpin DA Tili E Collard CD. Spinal cord injury after thoracic endovascular aortic aneurysm repair. Can J Anaesth. (2017) 64(12):1218–35. 10.1007/s12630-017-0974-1 PMC5954412 29019146

[13] Protack CD Smith A Moennich LA Hardy D Lyden SP Farivar BS. Midterm outcomes of subclavian artery revascularization in the setting of thoracic endovascular aortic repair. J Vasc Surg. (2020) 72(4):1222–8. 10.1016/j.jvs.2019.11.049 32093914

[14] Xue Y Sun L Zheng J Huang X Guo X Li T The chimney technique for preserving the left subclavian artery in thoracic endovascular aortic repair. Eur J Cardiothorac Surg. (2015) 47(4):623–9. 10.1093/ejcts/ezu266 25009212 PMC4358408

[15] Zhu Y Guo W Liu X Jia X Xiong J Wang L. The single-centre experience of the supra-arch chimney technique in endovascular repair of type B aortic dissections. Eur J Vasc Endovasc Surg. (2013) 45(6):633–8. 10.1016/j.ejvs.2013.02.016 23540806

[16] Percy ED Sabe AA. Commentar: A new chapter in chronic type B aortic dissection: balloon fracture fenestration and remodeling. J Thorac Cardiovasc Surg. (2020) 17(20):22–3. 10.1016/j.jtcvs.2020.10.036 33190875

[17] Shen Y Zhang S Zhu G Chen Y Chen Z Jing Z Risk factors ofdistal segment aortic enlargement after complicated type B aortic dissection. J Interv Med. (2019) 2(4):154–9. 10.1016/j.jimed.2019.10.003 PMC8562164 34805893

[18] Chang H Wang Y Liu B Wang W Li Y. Endovascular repair for acute type B aortic dissection with unfavorable proximal landing zone. Ann Thorac Surg. (2022) 113(2):545–53. 10.1016/j.athoracsur.2021.02.092 33819473

